# The Keighley Cottage Hospital

**Published:** 1887-10-15

**Authors:** E. Pringle

**Affiliations:** Keighley


### THE KEIGHLEY COTTAGE HOSPITAL.

The great disproportion of net proceeds to the expenses of the concert for the Keighley Cottage Hospital admits of a simple explanation. Arrangements had been made for an out-door concert (brass band engaged), but the weather proving unfavourable, the concert had to be held under cover, and, of course, the attendance was nothing like what it was on a previous occasion when the weather was propitious, and a substantial sum—between £30 and £40—was handed over to the Hospital Fund.

Keighley, Oct. 6, 1887. E. Pringle.

